# FOODLIT-Trial: Protocol of a Randomised Controlled Digital Intervention to Promote Food Literacy and Sustainability Behaviours in Adults Using the Health Action Process Approach and the Behaviour Change Techniques Taxonomy during the COVID-19 Pandemic

**DOI:** 10.3390/ijerph19063529

**Published:** 2022-03-16

**Authors:** Raquel Rosas, Filipa Pimenta, Isabel Leal, Ralf Schwarzer

**Affiliations:** 1 WJCR-William James Center for Research, ISPA-Instituto Universitário, 1149-041 Lisboa, Portugal; filipa_pimenta@ispa.pt (F.P.); ileal@ispa.pt (I.L.); 2 Department of Psychology, Freie Universität Berlin, 14195 Berlin, Germany; ralf.schwarzer@fu-berlin.de; 3 Department of Clinical, Health, and Rehabilitation Psychology, SWPS University of Social Sciences and Humanities, 53-238 Wroclaw, Poland

**Keywords:** food literacy, behaviour change, Behaviour Change Techniques Taxonomy, Health Action Process Approach, randomised controlled trial, COVID-19

## Abstract

Dietary quality and sustainability are central matters to the international community, emphasised by the burden of the COVID-19 pandemic. To promote healthier and more sustainable food-related practices, the protocol of a web-based intervention to enhance adults’ food literacy is presented. The FOODLIT-Trial is a two-arm, parallel, experimental, and single-blinded randomised controlled trial delivered over 11 weeks. Based on the Food Literacy Wheel framework and supported by the Health Action Process Approach (HAPA) and the Behaviour Change Techniques Taxonomy, weekly content with customised behaviour change techniques (experimental group) is hypothesised to be more effective to promote food behaviour change when compared to a single-time and non-customised delivery of food-related international guidelines, with no theoretically informed approaches (comparison group). Primary outcome is food literacy, including food-related knowledge, skills, and behaviours, assessed with the FOODLIT-Tool; a secondary outcome includes psychological mechanisms that efficaciously predict change in participants’ food literacy, measured with HAPA-driven items. Enlisted through online sources, participants will be assessed across five time points (baseline, post-intervention, and 3-, 6-, and 9-month follow-ups, i.e., T0–T4). A randomisation check will be conducted, analyses will follow an intention-to-treat approach, and linear two-level models within- (T0–T4) and between-level (nested in participants) will be computed, together with a longitudinal mediation analysis. If effective, the FOODLIT-Trial will provide for a multidimensional and cost-effective intervention to enable healthier and more sustainable food practices over the long term.

## 1. Introduction

Both adequate nutrition and worldwide environmental sustainability are strongly sustained by global food systems. In the last decades, imposed by diverse anthropogenic sources, such as growing population and uncertainty of global economy, food systems have been facing major alterations that have deeply impacted food consumption behaviours [1,2,3]. Intricately linking human health and sustainability, food consumption patterns represent one of the greatest challenges of this century. Trending unhealthy meal patterns, often driven by needs of convenience and inadequate accessibility to nutritious foods, are characterised as high in caloric value, excessively processed, and rich in animal source foods [3,4]. Leading to over 2 billion adults with overweight or obesity and a global prevalence of non-communicable diseases such as diabetes, unhealthy diets pose a greater risk to morbidity and mortality than those of unprotected sex, alcohol, tobacco, and drug use combined [3,5]. Moreover, with the global public health pandemic of COVID-19, food consumption behaviours are demonstrating an increased pattern of unhealthier diets during home confinements and other related restrictions across diverse countries [6].

Additional to increasing the burden of food-related diseases, these unhealthy dietary trends also play a crucial role in environmental degradation [7,8]. Food regimes identified as lose–lose diets—characterised by being both unhealthy and environmentally unsustainable—are not only described as high in saturated fats, added sugars, and red meats, but also represent a higher environmental burden, being associated with the transformation of natural ecosystems into croplands and threatening biodiversity with species’ extinction [9]. With 40% of global land occupied by agriculture, and food production being accountable for up to 70% of freshwater use and 30% of worldwide greenhouse-gas emissions, a change in the global food system is needed to minimise its impact on both human health and environmental sustainability [3,10,11,12].

A shift towards improved nutrition and more sustainable food systems has been a concern to the international community, represented by global agendas such as the Sustainable Development Goals integrated within the 2030 Agenda [13], its Food Systems Summit [14], and the Decade of Action on Nutrition [15]. However, this shift will not thrive without a simultaneous bottom-up transformation; it is crucial that people change how they view, understand, and engage with food systems, ultimately changing their food-related knowledge, competencies, and behaviours—that is, their food literacy [3,4,16,17].

### 1.1. Food Literacy

Designated as crucial to protect the quality of diets across the lifespan, food literacy has been gaining prominence across research, practice, and policy during the last decade [18,19,20,21,22,23]. Generally seeking to improve nutrition knowledge and food-related skills, most programmes and interventions developed within the scope of food literacy either (i) exclusively feature nutrition information [24,25,26], (ii) are targeted towards younger populations and often developed in an educational context [27,28,29,30,31], and/or (iii) narrowly focus on preparation or cooking skills, not emphasising other food-related competencies (e.g., planning, acquisition) [21,25,28,32]. More importantly, current interventions do not provide for knowledge to face the complexity of today’s food environment, nor the competencies to deal with it and navigate within aiming for healthier food patterns; consequently, food-related behaviour change is limited [32].

Acknowledging the intertwined relation among food system stakeholders and individuals’ food literacy, and its relevance in order to tackle major challenges concerning global sustainability, this team developed the Food Literacy Wheel (FLW) [16] and the FOODLIT-Tool [17]. The first is a conceptual and empirical framework of food literacy, comprehending not only the set of food-related knowledge, competencies, and behaviours but also its determinants (such as convenience and practicality, time and financial management, access to food information, and professionals’ unpreparedness on food-related expertise) and influential factors (psychological and learning surroundings, policy and industry settings, sustainability and social contexts, among others). The second concerns a validated and reliable instrument to assess the food literacy of adults based on the FLW; this quantitative measure allows for its own tailoring to diverse contexts and intends to evaluate one’s food literacy, its determinants, and influential factors, as a resource to promote behaviour change towards more healthier and sustainable food habits.

Aiming to make a contribution for the development of food-related competencies, attainment of healthier eating habits and achievement of more sustainable practices within one’s diet, the FOODLIT-Trial will integrate both the FLW and the FOODLIT-Tool on a digital intervention to promote food literacy and sustainability behaviours in adults.

### 1.2. Digital Interventions to Promote Behaviour Change

The use of technology within the daily life of developed countries’ population has gained particular relevance in recent years, being even more emphasised by the current COVID-19 global pandemic. With almost 90% of European households having online access and more than 70% adults affirming the use of online resources on an everyday basis, studies conducting digital interventions aiming for behaviour change have become widespread [33,34,35]. Particularly in the scope of health promotion, food consumption has been one of the most mainstream topics for the use of digital technologies; accounting for daily activities, the potential for food-related behaviours (such as purchasing, cooking, or eating) to be changed through digital solutions, such as web-based self-guided programmes and smartphone applications, is significantly appealing [35,36]. However, with the increase in digital interventions for the promotion of food-related healthier and sustainable behaviours, various trends have emerged. Within the theme of food sustainability, targeted behaviours have mainly focused on the reduction of food waste [37,38,39,40]; food-related competencies, purchasing, and cooking have been the most recurrent aimed behaviours [41,42,43]. The predominance of programmes targeted at younger populations [44,45] or specific to clinical conditions [46,47] is also notorious. Particularly concerning food literacy, the use of digital tools to promote food-related knowledge, competencies, and behaviours is still taking its first steps; either featuring technology or not, the prevalence of a younger target across food literacy interventions and programmes is evident [22,31,32,48]. More recently, however, the adult population has been targeted in research-based interventions [49,50,51,52], and digital resources remain scarce in the field.

Another noticeable characteristic of digital interventions to promote for healthy, sustainable, and knowledgeable food-related behaviours is the lack of clear theoretical backdrop to sustain behavioural change. The majority of these studies are scarcely grounded on a behavioural change theory [37,38]; most report an increase in participants’ awareness but do not explore longitudinal and evidence-based behaviour change [35]. Limitations of previous studies include lack of baseline data, lack of control or comparisons group, and lack of longitudinal follow-up data [35,43,49].

Addressing the promotion of healthier and more sustainable food-related knowledge, competencies, and behaviours through a digital and online intervention, the FOODLIT-Trial is grounded in the Health Action Process Approach (HAPA) [53,54] and applies behaviour change techniques from a consensual taxonomy (Behaviour Change Techniques Taxonomy (BCTT)) [55], aiming to lead to effective and sustained food behaviour change.

### 1.3. Study Objectives and Hypothesis

This study presents the detailed research protocol of a randomised controlled trial (RCT) to assess the efficacy of a web-based intervention in enhancing adults’ food literacy, using (i) digital evidence-based resources, (ii) behavioural change techniques from the BCTT [55], and (iii) the HAPA framework [53,54] as a theoretical backdrop.

The study’s primary objective is to evaluate whether the developed digital intervention is effective in improving food-related knowledge, competencies, and behaviours, based on the FLW [16] and evaluated with the FOODLIT-Tool [17]. Potential differences in participant’s food literacy over time will also be assessed with a longitudinal design. We hypothesise that the use of a web-based intervention combined with behavioural change strategies (customised to each food-related skill) will be more effective to enhance food literacy than the approach used with the comparison group (single-time delivery of non-customised food-related national and international guidelines, without any additional theoretically informed, evidence-based behaviour change approaches). The second objective is to understand the intervention performance, by evaluating which psychological mechanisms, such as self-efficacy, planning, and action control [53,54], efficaciously determine change in participants’ food literacy. It is hypothesised that HAPA-derived mechanisms will significantly mediate the participants’ outcomes concerning food literacy.

## 2. Methods and Analysis

### 2.1. Trial Design

**Figure 1:**
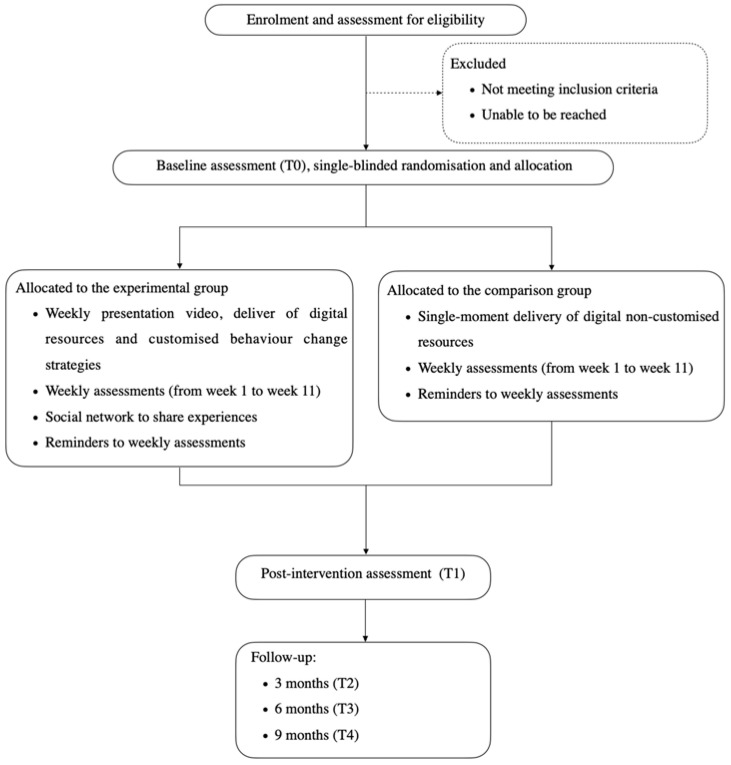
Flowchart of the FOODLIT-Trial intervention, including both experimental and comparison groups.

The FOODLIT-Trial is a two-arm (allocation ratio 1:1), parallel, experimental, and single-blinded randomised controlled trial for Portuguese adults (Figure 1). The web-based intervention is delivered over 11 weeks, where each week is themed with content either according to the FLW framework or to the HAPA model.

In conformity to the week’s thematic, each ability, skill, and behaviour is matched with a behavioural change strategy to facilitate its implementation [55]. All measures will be assessed at five time points: baseline (T0), to measure baseline characteristics, pre-intervention, before randomised allocation, and prior to the trial’s first week; post-intervention (T1); one week after the 11-week intervention delivery; and at follow-up times 3, 6, and 9 months after the intervention (T2, T3, and T4, respectively).

This protocol adheres to the Consolidated Standards of Reporting Trials [56] guidelines for randomised controlled trials.

### 2.2. Ethical Approval

As part of a major project titled FOODLIT-PRO: Food Literacy Project, this study was approved by the Ethics Committee of Ispa—Instituto Universitário (ref. D/002/03/2018). The FOODLIT-Trial was developed according to the Declaration of Helsinki, followed the deontological norms and ethical principles of the Order of Portuguese Psychologists [57], and adhered to General Data Protection Regulation [58]. This protocol was approved and registered by ClinicalTrials.gov (NCT04806074).

### 2.3. Participants and Recruitment

Considering its web-based format, FOODLIT-Trial’s potential participants will represent a sample of convenience and snowballing, and will be reached and enlisted through online sources. Online reach out will be made by using both advertisements in social media websites according to the researcher’s network (Instagram and Facebook, Meta Platforms: Cambridge, MA, USA) and a developed website for participants’ enrolment. During the recruitment stage, potential participants will be informed that trial participation will entail compensation in order to acknowledge their time and effort dedicated to the study.

An a priori power analysis was conducted with G*Power (v. 3.1), and a minimum sample size of 28 was necessary in order to detect a medium effect size (Cohen’s d = 0.50) at the 5% level of significance with 95% power, with the assumption of the non-violation of sphericity (non-sphericity correction ε = 1) considering the trial’s repeated-measures design. Given a potential attrition rate of 50% due to the digital nature of the trial, its duration, and required weekly assessments, a minimum of 56 participants will be recruited.

Participants for the FOODLIT-Trial must (i) be adults aged 18 years or older, (ii) be able to understand and read Portuguese, (iii) have availability to engage in the 11-week trial, and have internet access that allows for their engagement, (iv) be responsible for, at least, one out of four tasks in their food routine (encompassing choice and decision, selection and acquisition, preparation, and cooking, according to [18]). Potentially eligible participants will be invited to the trial through an online information sheet, and will be provided with the consent form; if eligible, the baseline questionnaire (T0) will be made available and delivered online. Additionally, all participants will be asked to complete a sociodemographic questionnaire aiming to collect self-reported data concerning sociodemographic and health-related characteristics (e.g., sex, age, educational level, diagnosed diseases, height, weight).

### 2.4. Randomisation and Blinding

Consenting participants meeting inclusion criteria will be randomised and allocated to either the experimental group (EG) or the comparison group (CG), following the baseline period. At baseline, each participant will create a unique code (based on the name’s initials and year of birth) to allow for longitudinal correspondence along the different time points. Randomisation will then be performed using a computer-generated random 1:1 allocation list. Knowledgeable concerning the specifications of the trial arms in the consent form, all participants will also be informed that both groups will (i) be contacted weekly to take part in every assessment, (ii) receive the same online reminders through digital sources (email and WhatsApp, Meta Platforms: Mountain View, California), and (iii) be featured in the compensation mechanism. As such, randomisation results will be concealed from participants at all moments. It will not be possible to apply this to the research coordinator (RR), given her responsibility to create and deliver the weekly customised resources to the EG. Thus, the FOODLIT-Trial’s allocation will be single-blinded for its participants.

### 2.5. Intervention

The FOODLIT-Trial is an online-enabled intervention to promote food literacy and food sustainability practices delivered with digital evidence-based resources in multiple formats, based on theoretically informed behaviour change approaches, and made available through mobile phone, tablet, and computer. The intervention will include weekly reminders for participants to evaluate their food-related knowledge, competencies, and behaviours, and assess related psychological mechanisms associated with behaviour change. Experimental and comparison group specifications are described below.

#### 2.5.1. Experimental Group

Participants allocated to the EG will receive weekly information concerning a specific theme through digital sources such as videos, infographics, and web-directed links. The 11-week intervention is designed according to (a) the food-related knowledge, competencies, and behaviours belonging to the core of the FLW conceptual and empirical model, and (b) the psychological mechanisms within the HAPA framework. As shown in Table 1, each week entails not only a set of skills, behaviours, and/or mechanisms that feature the above mentioned theoretical and empirical frames, but also a customised, well-defined, and identifiable technique from BCCT to prompt participants’ food-related behaviour change.

Participants in the EG will receive weekly (A) evidence-based and customised information related to a specific skill, behaviour, and/or mechanism, from sources such as national and international guidelines—namely, the Portuguese Directorate-General for Health and the EAT-Lancet Commission on Food, Planet, Health; (B) designated tasks based on each behaviour change technique and related to the week’s thematic; (C) a short introductory video, featuring the research coordinator, briefly mentioning the week’s thematic and alerting to the week’s assessment; (D) notifications reminding the need to respond to the week’s questionnaire (two days before the end of the week and the day of the due date for questionnaire response) and the corresponding link leading to the week’s questionnaire. All materials, except for the weekly questionnaires, will be stored in a purposely created website, allowing for participants to revisit previous weeks’ resources (if desired).

Shown in Table 2A is two-week example of (A) a customised infographic, (B) its associated task based on a behaviour change strategy, and (C) its corresponding items.

#### 2.5.2. Comparison Group

Participants allocated to the active CG will receive a single-time and non-customised delivery of the same food-related national and international guidelines. There will be no theoretically informed approach and no behaviour change techniques, and the delivered content will generically regard nutritious eating and food-related habits. No digital presence of the research coordinator will be featured to the CG (that is, no weekly introductory videos will be sent to this cohort). Additionally, to the (A) single-time (but non-customised) delivery of informative guidelines from the same entities, the CG will receive (D) the identical notifications serving as reminders for the weekly questionnaires (identical to the questionnaires delivered to the EG). Similarly to the EG, the guidelines will be stored in a specifically designed website, providing for uninterrupted access.

### 2.6. Adherence and Strategies to Minimise Drop-Out

To encourage intervention adherence and engagement, weekly reminders will be sent to participants of both EG and CG via email and/or WhatsApp. At the beginning of each week, the reminder will be sent via WhatsApp, notifying participants that a new week of FOOLIT-Trial is starting and that the weekly welcoming email was sent; for the EG, a link enabling the access to the week’s resources (A, B, and C) will be featured, while CG participants will be reminded of the access to where guidelines are stored (non-customised A). Two days before the end of each week, an email will be sent in the morning with the link leading to the week’s questionnaire; a notification through WhatsApp will be delivered later that day reminding that the link for questionnaire response is already available. At the due date for the weekly response, a final reminder will be sent through WhatsApp, indicating that the questionnaire will be available until the end of that day.

Additionally, as a strategy to minimise drop-out and to prompt continuous engagement, compensation will be featured within the FOODLIT-Trial. Compensation will entail the following randomised allocations of gift cards to participants for grocery shopping: (i) one gift card at the end of the 11-week intervention (with a credit of 50 EUR), (ii) a gift card at the end of the first and second follow-up (T2 and T3; a total of two gift cards with a credit of 25 EUR each), and (iii) two gift cards at the end of the last follow-up (T4; each gift card with a credit of 50 EUR).

### 2.7. Outcomes

#### 2.7.1. Primary Outcome Measure

Considering FOODLIT-Trial’s first aim, the primary outcome to be assessed is food literacy. Food-related knowledge, competencies, and behaviours will be assessed with the FOODLIT-Tool [17] at baseline (T0), during the 11-week intervention (with the items distributed across the theme for the week; Table 1), post-intervention (T1), and at all follow-ups (T2, T3, and T4). These longitudinal assessments will evaluate participants’ food literacy according to the five dimensions portrayed in the instrument (Culinary Competencies, Production and Quality, Selection and Planning, Environmentally Safe, and Origin) and based on the FLW [16]. These include (i) theoretical knowledge, such as knowing various types of food preservation suitable to different foods (item 10); (ii) practical competencies, as interpreting food labels to select adequate foods (item 19); and (iii) food habits and behaviours, such as eating foods according to their seasonality (item 22). All items are assessed with a four-point Likert-type response scale, concerning either frequency (0—*never*; 1—*sometimes*; 2—*frequently*; 3—*always*) or agreement (0—*completely disagree*; 1—*disagree*; 2—*agree*; 3—*completely agree*).

#### 2.7.2. Secondary Outcome Measure

Aiming to explore intervention performance, FOODLIT-Trial’s second aim is to evaluate which psychological mechanisms efficaciously predict change in participants’ food literacy. To achieve this objective, psychological mechanisms derived from the HAPA—including risk perception, outcome expectancies, self-efficacy, planning, and action control—will also be assessed at similar time points (from T0 to T4). All measures to evaluate HAPA constructs are adapted from Sniehotta, Scholz, and Schwarzer [59] and are specific to food literacy, depicting eating according to one’s needs as the intended behaviour. All items are also assessed with four-point Likert-type response scales, regarding agreement (0—*totally disagree*; 1—*disagree*; 2—*agree*; 3—*totally agree*) and possibility (0—*very unlikely*; 1—*unlikely*; 2—*likely*; 3—*very likely*).

## 3. Statistical Analyses

### 3.1. Randomisation Check, Drop-Out Analyses, and Intention to Treat

A randomisation check will address equal distributions of all baseline measures of all primary and secondary outcomes and covariates across conditions using multivariate analyses of variance interval–scale data, and chi-square tests for nominal and ordinal-scale data. Analyses will be carried out in an intention-to-treat manner, accounting for missing values using the full information maximum likelihood approach [60]. Drop-out analyses will test baseline differences between continuers and non-continuers in all variables using t-tests, chi-square tests, or logistic regression.

### 3.2. Hypotheses Tests for Intervention Effects

Linear two-level models with five time points (T0, T1, T2, T3, T4; within-level) nested in participants (between-level) will be computed. For each outcome measure, time (linear day trend, centred at 0) x experimental condition (0 = comparison condition; 1 = intervention condition) interactions will be estimated. Moreover, grand-mean centred covariates (e.g., sex, age) will be added as between-level predictors. The linear time trend and the linear time trend x experimental condition interaction will be modelled as fixed effects.

### 3.3. Examining Intervention Mechanisms

To explore the assumptions of the HAPA, a series of longitudinal mediation analyses will be conducted using manifest or latent path analyses. Experimental condition will be specified as a dummy-coded independent variable, proposed cognitive mechanisms as mediators, and food literacy factors as the outcomes (with or without control for respective baseline assessments). Because of the flexible conceptual framework of HAPA, reasonable time points (T1–T4) will be explored to identify the most useful mediators (e.g., self-efficacy, outcome expectancies, behavioural intention, planning) within the entire time span of the study. Bias-corrected bootstrap confidence intervals (95%) of direct and indirect effects will be generated by bootstrapping with 5000 re-samples.

## 4. Dissemination Plan

The study protocol is the first publication of this RCT. Findings of this RCT will be published in peer-reviewed international journals and at national and international conferences. Dissemination of results in journals will comply with CONSORT guidelines. Important protocol modifications will be reported.

## 5. Conclusions

By introducing the research protocol of a RCT that aims to evaluate the efficacy of a digital intervention to promote adults’ food literacy, this study highlights not only the use of web-based resources to tackle food-related competencies and behaviours, but also addresses the need to design and apply a trial based on strong theoretical foundations linked to health behaviour change. We hypothesise that the support allowed by the delivery of digital materials entailing behavioural change strategies customised to food literacy-related information will improve food knowledge, competencies, and behaviours. A secondary hypothesis is that mechanisms acknowledged as part of a theoretical background to promote behaviour change will mediate these food literacy outcomes. To achieve the hypothesised outcomes, this team developed an 11-week plan that (A) gathers evidence-based resources based on national and international guidelines, (B) designates specific and diversified tasks based on behaviour change techniques, (C) provides for a multiple thematics, and (D) shares online notifications.

Presenting the first known randomised digital intervention to integrate behavioural strategies, based on a validated taxonomy and a theoretical framework of behaviour change in the field of food literacy, the FOODLIT-Trial intends to contribute to the promotion of healthier and more sustainable food habits during a global public health pandemic. With growing evidence on the impact of the COVID-19 pandemic on consumers’ food patterns and worldwide food security [2,61,62], it is urgent to provide for mechanisms that promote positive change on food-related competencies and behaviours, while providing for strategies that guide one’s navigation within this transformative food system. Accounting for a specific web-based platform for the delivery of digital resources and integrating online communication throughout the intervention, the FOODLIT-Trial transforms extensive international recommendations into thematic weekly challenges with the expectation to advocate for more informed food knowledge and more adequate and sustainable eating habits in adult population. If effective, this intervention—along with its assessments of the FOODLIT-Tool and its conceptual basis from the FLW—has the potential to be adapted and applied across multiple professional contexts, allowing for a digital cost-effective resource that promotes healthier and more sustainable food habits according to international guidelines.

## Figures and Tables

**Table 1:** Description of the experimental group intervention, including (i) the weekly thematic; (ii) its correspondent frameworks, including the Food Literacy Wheel (FLW) and the Health Action Process Approach (HAPA); (iii) the instruments used for weekly assessment, entailing either items from the FOODLIT-Tool or the HAPA; and (iv) the identification of the each behaviour change technique (BCT) used across all weeks, customised to the thematic’s content.

Week	Framework	Instruments	Behaviour Change Techniques
Thematic	Variables or dimensions	Dimensions and/or items	BCTs title
**Week 1**	**HAPA**	**HAPA**	**Total: 3 BCTs**
Pre-intenders	Action self-efficacy	Five items	(15.1) Verbal persuasion about capability
	Risk perception	Three items	(5.1) Information about health consequences
	Outcome expectancies	Nine items	(9.3) Comparative imagining of future outcomes
**Week 2**	**FLW**	**FOODLIT-Tool**	**Total: 2 BCTs**
Origin and conservation	Choice and acquisition	*Origin* Items 17 and 18	(4.1) Instruction on how to perform the behaviour
	Preserve and analyse	*Culinary competencies* Item 10	(4.1) Instruction on how to perform the behaviour (6.1) Demonstration of the behaviour
**Week 3**	**FLW**	**FOODLIT-Tool**	**Total: 4 BCTs**
Prepare and adapt	Cooking Skills	*Culinary competencies* Item 1	(1.4) Action planning (4.1) Instruction on how to perform the behaviour (6.1) Demonstration of the behaviour
		Item 2	(4.1) Instruction on how to perform the behaviour
		Item 3	(15.1) Verbal persuasion about capability
**Week 4**	**FLW**	**FOODLIT-Tool**	**Total: 3 BCTs**
Cooking	Cooking Skills	*Culinary competencies* Item 4	(6.1) Demonstration of the behaviour
		Item 8	(1.1) Goal setting (behaviour) (4.1) Instruction on how to perform the behaviour (6.1) Demonstration of the behaviour
**Week 5**	**FLW**	**FOODLIT-Tool**	**Total: 1 BCT**
Choice and selection	Choice and acquisition	*Selection and planning* Item 20	(4.1) Instruction on how to perform the behaviour *(in both items)*
		Item 21	
**Week 6**	**HAPA**	**HAPA**	**Total: 3 BCTs**
Intenders	Maintenance self-efficacy	Six items	(15.3) Focus on past success
	Action planning	Five items	(1.4) Action planning
	Coping planning	Six items	(1.2) Problem solving
**Week 7**	**FLW**	**FOODLIT-Tool**	**Total: 3 BCTs**
Nutrition and intake	Cooking Skills	*Culinary competencies* Item 5	(6.1) Demonstration of the behaviour
	Preserve and analyse	*Selection and planning* Item 16	(2.4) Self-monitoring of outcome(s) of behaviour
	Choice and acquisition	Item 19	(6.1) Demonstration of the behaviour
	Search and plan	Item 24	(4.1) Instruction on how to perform the behaviour
**Week 8**	**FLW**	**FOODLIT-Tool**	**Total: 4 BCTs**
Planning and cooking pleasure	Cooking skills	*Culinary competencies* Item 9	(5.6) Information about emotional consequences (10.4) Social reward
	Search and plan	*Selection and planning* Item 25	(4.1) Instruction on how to perform the behaviour *(in both items)*
		Item 26	(6.1) Demonstration of the behaviour *(in both items)*
**Week 9**	**FLW**	**FOODLIT-Tool**	**Total: 2 BCTs**
Hygiene and safety (within production and kitchen)	Preserve and analyse	*Environmentally safe* Item 11	(4.1) Instruction on how to perform the behaviour (6.1) Demonstration of the behaviour
		*Production and quality* Item 12	(4.1) Instruction on how to perform the behaviour *(in all items)* (6.1) Demonstration of the behaviour *(in all items)*
		Item 13	
		Item 14	
**Week 10**	**FLW**	**FOODLIT-Tool**	**Total: 2 BCTs**
Local and seasonal	Preserve and analyse	*Environmentally safe* Item 15	(5.3) Information about social and environmental consequences
	Search and plan	Item 22	(4.1) Instruction on how to perform the behaviour *(in both items)*
		Item 23	
**Week 11**	**HAPA**	**HAPA**	**Total: 3 BCTs**
Actors	Recovery self-efficacy	Three items	(8.7) Graded tasks
	Action control	Six items	(1.6) Discrepancy between current behaviour and goal (2.3) Self-monitoring of behaviour

**Table 2:** Example of the FOODLIT-Trial’s experimental group Week 4 (themed Cooking) and Week 11 (themed Actors), entailing (A) customised infographics; (B) its associated tasks, presented within the infographics and reflecting the behaviour change strategies applied; and (C) corresponding items, to be responded to before the end of the week.

Week 4—Cooking
Customised infographics (A) and its associated tasks (B), reflecting the behaviour change and strategies applied.
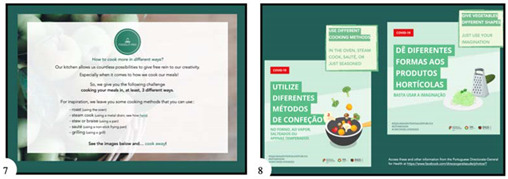
Corresponding items (C), to be responded to before the end of the week. Item 4 *I use kitchen equipment and utensils (e.g., oven, blender) efficiently.* Four-point Likert-type response scale (0—*never;* 1—*sometimes;* 2—*frequently;* 3—*always*). Item 8 *I cook in different ways (e.g., stewing, baking).* Four-point Likert-type response scale (0—*never*; 1—*sometimes*; 2—*frequently*; 3—*always*).
**Week 11—Actors**
Customised infographics (A) and its associated tasks (B), reflecting the behaviour change strategies applied.
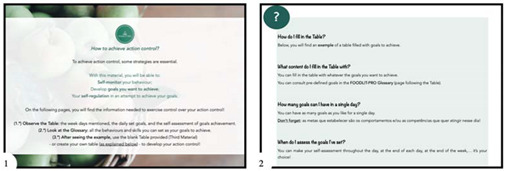
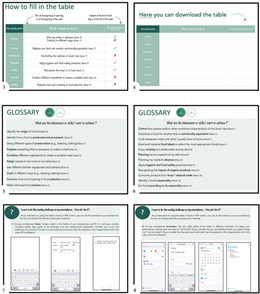
Corresponding items (D), to be responded to before the end of the week. **Recovery self-efficacy** (three items) *I believe that I could return to having a diet adequate to my needs, even if:* (1) *I had spent a few days without doing so.* (2) *I had spent many days without doing so.* (3) *I had spent a few weeks without doing it.* **Action control** (six items) (1) *I have evaluated regularly when, where and how I am making an adequate diet suited to my needs.* (2) *I have assessed my behaviour daily to check if I am having an adequate diet.* (3) *I am always aware of the diet that is adequate to my needs.* (4) *I have always in mind the intention to make a diet adequate to my needs.* (5) *I have worked hard to have a diet that meets my needs on a regular basis.* (6) *I have been making the effort to have an adequate diet as much as I intend to.*

## Data Availability

Not applicable.
